# Genomic Selection in Sugarcane: Current Status and Future Prospects

**DOI:** 10.3389/fpls.2021.708233

**Published:** 2021-09-27

**Authors:** Channappa Mahadevaiah, Chinnaswamy Appunu, Karen Aitken, Giriyapura Shivalingamurthy Suresha, Palanisamy Vignesh, Huskur Kumaraswamy Mahadeva Swamy, Ramanathan Valarmathi, Govind Hemaprabha, Ganesh Alagarasan, Bakshi Ram

**Affiliations:** ^1^Division of Crop Improvement, ICAR-Sugarcane Breeding Institute, Coimbatore, India; ^2^CSIRO (Commonwealth Scientific and Industrial Research Organization), St. Lucia, QLD, Australia; ^3^Division of Crop Production, ICAR-Sugarcane Breeding Institute, Coimbatore, India

**Keywords:** sugarcane, genomic selection, pre-breeding, genetic base-broadening, multi-environment trials, genomic estimated breeding value

## Abstract

Sugarcane is a C4 and agro-industry-based crop with a high potential for biomass production. It serves as raw material for the production of sugar, ethanol, and electricity. Modern sugarcane varieties are derived from the interspecific and intergeneric hybridization between *Saccharum officinarum, Saccharum spontaneum*, and other wild relatives. Sugarcane breeding programmes are broadly categorized into germplasm collection and characterization, pre-breeding and genetic base-broadening, and varietal development programmes. The varietal identification through the classic breeding programme requires a minimum of 12–14 years. The precise phenotyping in sugarcane is extremely tedious due to the high propensity of lodging and suckering owing to the influence of environmental factors and crop management practices. This kind of phenotyping requires data from both plant crop and ratoon experiments conducted over locations and seasons. In this review, we explored the feasibility of genomic selection schemes for various breeding programmes in sugarcane. The genetic diversity analysis using genome-wide markers helps in the formation of core set germplasm representing the total genomic diversity present in the *Saccharum* gene bank. The genome-wide association studies and genomic prediction in the *Saccharum* gene bank are helpful to identify the complete genomic resources for cane yield, commercial cane sugar, tolerances to biotic and abiotic stresses, and other agronomic traits. The implementation of genomic selection in pre-breeding, genetic base-broadening programmes assist in precise introgression of specific genes and recurrent selection schemes enhance the higher frequency of favorable alleles in the population with a considerable reduction in breeding cycles and population size. The integration of environmental covariates and genomic prediction in multi-environment trials assists in the prediction of varietal performance for different agro-climatic zones. This review also directed its focus on enhancing the genetic gain over time, cost, and resource allocation at various stages of breeding programmes.

## Introduction

Sugarcane is an important agro-based industrial crop cultivated in tropical and sub-tropical regions; it serves as a raw material for the production of sugar, bioethanol, and bioenergy (Hoang et al., 2015). Globally, sugarcane is cultivated in 28.19 million hectares which produces 2059.74 million tonnes of canes annually with average productivity of 72.80 t ha^−1^ (FAOSTAT, 2019). As a C4 plant, it is a high biomass producing crop generating around 279 million tonnes of lignocellulosic biomass residues, i.e., leaves and bagasse, per year worldwide (Chandel et al., 2012). The adoption of new varieties has enhanced the cane and sugar yield in India, Australia, Brazil, and South Africa (Burnquist et al., 2010; Ram and Hemaprabha, 2020; Schmitz et al., 2020). Modern breeding approaches, such as genomic selection involving inter and multi-disciplinary approaches (Crossa et al., 2017), are required to further enhance the genetic gain for cane yield and sugar yield.

**Figure 1:**
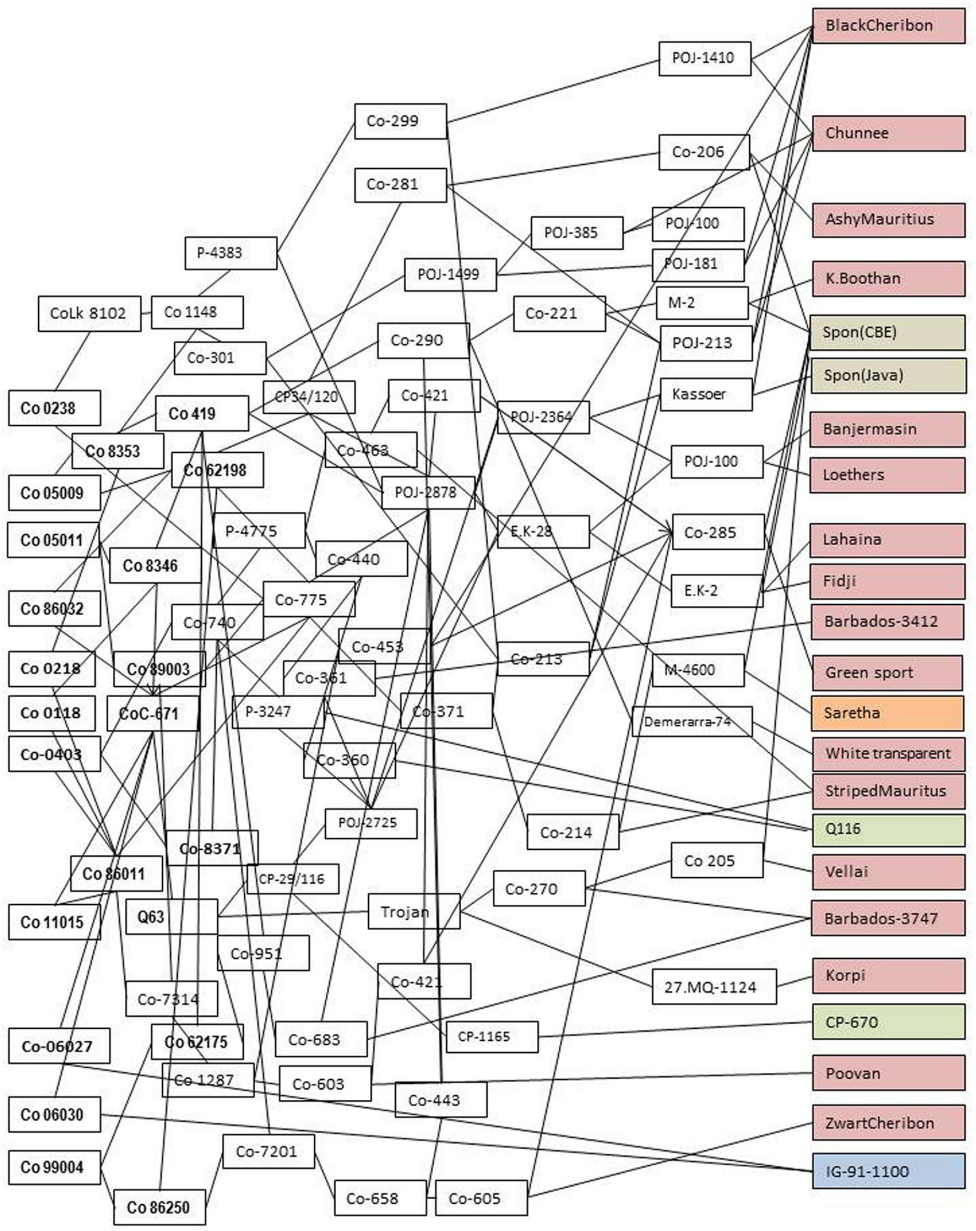
In-depth pedigree analysis of notified sugarcane varieties in India stipulating the total genetic variability present in the field. They are derived from the limited germplasm *viz.*, 17 clones of *S Saccharum officinarum* and its derivatives (pink color), one clone of *Saccharum barberi* (orange), two clones of *Saccharum spontaneum* (gray), one genetic stock IG91-1100 derived from intergeneric hybridization between sugarcane (CoC 772) and *Erianthus arundinaceus* (blue), and two foreign clones (green). The implementation of genomic prediction in sugarcane germplasm characterization and genomic selection in pre-breeding aids in introgression and augmentation of more favorable alleles, base-broadening of working germplasm, and finally deploying more number of favorable genes into the target environments.

Sugarcane is an exemplary crop that exploits the heterosis from the wild genetic resources. The modern cultivars are interspecific and intergeneric hybrids of *Saccharum officinarum, Saccharum spontaneum*, and other related genera (Bhat and Gill, 1985; Sreenivasan and Ahloowalia, 1987; Grivet and Arruda, 2002; Lekshmi et al., 2017). The basic clones of *Saccharum* species significantly contributed to the heterosis and genetic base diversification in sugarcane germplasm. The in-depth pedigree analysis of the modern sugarcane cultivars and breeding lines revealed that a limited number of germplasm and basic species were used in the varietal development programmes (Jackson, 2005). The pedigree analysis of Indian ‘Co’ varieties exhibited that only two *S. spontaneum* accessions *viz., S. spontaneum* CBE and *S. spontaneum* Java were used in the breeding programmes (Kumar et al., 2012). The pedigree analysis of notified thirteen varieties in India (Figure 1) depicts the narrow genetic base in cultivated varieties and these varieties are derived from the clones of a limited basic *Saccharum* species such as 17 accessions of *S*. *officinarum* and its derivatives, one *Saccharum barberi*, two *S. spontaneum*, and one *Erianthus arundinaceus* derived clone and two foreign clones. Though the diverse wild genetic resources were used in the pre-breeding and many breeding lines or genetic stocks were identified (Mohanraj and Nair, 2014; Nair et al., 2017), it might not have selected or captured all the desirable genes in the background of undesirable linkage drags. Hence, there is a requirement to take the advantage of the genome-wide markers and genomic selection for effective utilization of genetic resources in the sugarcane breeding programme.

Sugarcane breeding has been performed in three phases (Jackson, 2005). First is the crossing and selecting among the *S. officinarum* clones. Second is the interspecific hybridization and nobilization of canes. Third is the intermating or recurrent selection among the nobilized cane to develop the commercial canes. The genetic variability in the present working sugarcane germplasm is not fully exhausted or no yield plateau was observed in sugarcane (Edm et al., 2005), but there is evidence of slow genetic gain (Wei and Jackson, 2017). Additionally, new variability is continuously created through pre-breeding in many countries (da Silva, 2017; Nair et al., 2017; Cursi et al., 2021), yet the application of genomic selection in sugarcane is capable of capturing the entirety of the allelic variability and enhancing the generic gain (Deomano et al., 2020; Hayes et al., 2021; Voss-Fels et al., 2021; Yadav et al., 2021). The recent review on genomic selection in sugarcane highlighted the recurrent selection schemes (Yadav et al., 2020) and we reviewed the feasibility of genomic selection at various stages of sugarcane breeding programmes, such as germplasm evaluation and formation of core germplasm, pre-breeding and genetic base-broadening programmes, and multi-environmental trails of breeding lines.

## Genomic Selection in Sugarcane

The genetic gain for commercial sugar yield can be improved either by enhancing the cane yield or sucrose content, and both traits were reported to have a poor genetic correlation (Jackson, 2005). Both cane yield and sucrose were governed by many quantitative trait loci (QTL) or genomic regions (Hoarau et al., 2002; Ming et al., 2002; Aitken et al., 2008). The narrow-sense heritability and very high non-additive genetic variance for cane yield (Brown et al., 1968; Hogarth, 1971; Hogarth et al., 1981; Barbosa et al., 2005; Wei and Jackson, 2017), the selection of parents solely based on breeding values do not improve the genetic gain for cane yield (Wei and Jackson, 2017). The utility of genome-wide markers such as single nucleotide polymorphism (SNP) for prediction of breeding values of parents and genotypes through genomic selection approaches were demonstrated in other crops (Unterseer et al., 2014; Yu et al., 2014; Aitken et al., 2016; Pandey et al., 2017; Roorkiwal et al., 2018; Saxena et al., 2018; Li et al., 2019). The Diversity Array Technology (DArT), Axiom 345K SNP, and Affymetrix Axion 100K SNP arrays were now available (Heller-Uszynska et al., 2011; Berkman et al., 2014; Aitken et al., 2016; Song et al., 2016; You et al., 2018) and utilized for genomic prediction studies in sugarcane (Gouy et al., 2013; Deomano et al., 2020; Hayes et al., 2021; Voss-Fels et al., 2021; Yadav et al., 2021). The accuracy of genomic prediction or selection studies depends on many parameters such as robust statistical models, genome-wide markers, and parameters related to training and testing populations such as population structure, trait heritability, trait architecture, genetic diversity, and genetic relatedness (Meuwissen et al., 2001; Zhang et al., 2017; Berro et al., 2019).

### Genomic Prediction Models

The genomic prediction models are statistical models, which combines the pedigree data, genotypic data, phenotypic data, and environmental covariates to estimate the genomic estimated breeding values (GEBV) and enhance the prediction accuracies (Meuwissen et al., 2001; Rincent et al., 2012; Heslot et al., 2014; Malosetti et al., 2016; Monteverde et al., 2019). This is a multi-disciplinary approach involving computer science, genetics, quantitative genetics, statistics, bioinformatics, mathematics, physics, and machine learning to deduce the estimated breeding values (Crossa et al., 2017). The genomic prediction models also involve cross-validation to determine their accuracies (Burgueño et al., 2012; Rincent et al., 2012, 2017). Most of the statistical models, such as ridge-regression best linear unbiased prediction (RR-BLUP), BayesA, BayesB, BayesC, least absolute shrinkage and selection operator (LASSO), and genomic BLUP (GBLUP), estimate the additive genetic variance or breeding values (Wang et al., 2018b). In sugarcane genomic prediction studies, genomic models such as BayesA, BayesB, Bayesian LASSO, Bayesian GBLUP, and reproducing kernel Hilbert space (RKHS) were used to predict the commercial cane sugar (CCS) and cane yield in early and advance breeding experiments in Australia. The prediction accuracy (0.25–0.45) was highly encouraging under additive genomic models. The prediction accuracies for CCS in advanced clonal selection trials which was higher than the early clonal trials and, vigorous selection in early clonal trials did not affect the prediction accuracies of advanced selection trials. The reverse trend was observed for cane yield, wherein the prediction accuracies in advanced trials were less than that of early clonal trials (Deomano et al., 2020) and it was attributed to the competition effects in smaller plots (Jackson and McRae, 2001). Three additive genomic models namely GBLUP, genomic single step, and BayesR were tested against 3,984 breeding lines genotyped with 26K SNP, achieving satisfactory prediction accuracies (0.30–0.44) with 5% improvement for CCS and fiber without any improvement for cane yield due to competition effects in early breeding trials (Hayes et al., 2021).

The non-additive genetic variance significantly contributes to the heterosis in sugarcane and the low narrow-sense heritability is one of the causes for slow genetic gain to cane yield (Wei and Jackson, 2017). RKHS and extended GBLUP genomic models captures the non-additive genetic variance (Endelman, 2011; Jiang and Reif, 2015; Momen and Morota, 2018; Momen et al., 2018; Varona et al., 2018) and RKHS genomic model was used in sugarcane genomic selection (Gouy et al., 2013; Yadav et al., 2021). In simulation studies, genetic gain due to additive genomic model was almost double than the phenotypic selection and the genomic selection enhanced the rapid increase in frequencies of favorable alleles. Whereas, under the non-additive model, the genetic gain under genomic selection was still higher than the phenotypic selection and the success of the genomic selection depends on genomic breeding schemes, clonal population and stage of breeding programmes (Voss-Fels et al., 2021). In another study, extended GBLUP incorporated with additive, dominance, epistatic, and average genome-wide heterozygosity components to dissect the non-additive variance for cane yield, CCS, and fiber. The large portion of variance was attributed to dominance variance for cane yield and epistatic genetic variance for both cane yield and CCS. The epistatic genetic variance (additive × additive genetic variance) and variance due to genome-wide heterozygosity had accounted significant portion of genetic variance for both cane yield and CCS under additive-dominance-epistatic and additive-dominance-epistatic-genome-wide heterozygosity genomic models (Yadav et al., 2021).

Genomic prediction models are evolving science, and several modifications of the genetic relationship matrices were reported to enhance the prediction accuracies. The trait associated BLUP (taBLUP) make use of the trait-specific relationship matrix which captures the genetic variance of a locus, whose elements were derived based on the probability of a marker locus identical by descent and genetic variance of a trait. The genetic architecture was incorporated into the kinship matrix by assigning the suitable weight to the markers identified through genome-wide association studies (GWAS) and additionally improved by removing the duplicate trait-specific markers associated with the same (Zhang et al., 2010; Wang et al., 2018a). Further, the elements of kinship matrices were modified by assigning the markers to bins or real quantitative trait nucleotides identified in GWAS. In super BLUP (sBLUP), the maximum likelihood association between the traits and markers were enhanced by incorporating the elements of kinship matrices by the markers identified within a bin or real quantitative trait nucleotide identified in GWAS. Whereas, in compressed BLUP (cBLUP), the kinship matrices were derived by clustering the genotypes into groups and each group was treated as a random effect (Wang et al., 2018a). Further studies are required to understand the impact of sBLUP and cBLUP in the background of additive, dominance, epistatic and genome-wide heterozygosity models for improving the prediction accuracies in sugarcane.

### Genome-Wide Markers

One of the most commonly raised issues in the sugarcane genome analysis is that it exhibits hyperploidy. Hyperploidy is a major problem in SNP identification among polyploids, as it may lead to false SNP calling in crops like sugarcane and strawberry. The challenges in SNP identification and SNP selection criteria in polyploids were recently reviewed (You et al., 2018; Manimekalai et al., 2020). In recently formed polyploid genomes, such as sugarcane, the problem is 2-fold for SNP calling. On the other hand, there is a paucity of high-end algorithms and computational methods, as large numbers of the available SNP analyzing tools are not suitable for the polyploid genome. Two relevant kinds of technology gaps have been identified in the sugarcane SNP genotyping. The first is that of homoeologous SNPs required to differentiate from the true allelic SNPs in polyploid crops. For read alignment, Bowtie-2 and Burrows-Wheeler Aligner - Maximal Exact Matches (BWA-mem) identify the read alignments based on the number of mismatch scores (Clevenger et al., 2015) and Universal Network-Enabled Analysis Kit (UNEAK) SNP discovery pipeline is also included in the Trait Analysis by aSSociation, Evolution and Linkage - Genotyping by sequencing (TASSEL-GBS) (Elshire et al., 2011; Lu et al., 2013). The various advanced sequencing platforms have demonstrated their role in diploid/polyploid crops. The Illumina platform presents the most robust and cost-effective method of polyploid genome analysis through short-read sequencing, even though a longer read length is preferred to sequence genomes like sugarcane, which possess high levels of repetitive regions (You et al., 2018). Currently, PacBio offers long-read sequencing on an average read length of 15 kb through single-molecule real-time sequencing (Manimekalai et al., 2020). However, researchers from developing nations are not able to use PacBio or any other advanced platforms due to the higher sequencing costs. Cheaper sequencing costs would facilitate in-depth sequence coverage, which is a preferred strategy in outcrossing species. In diploids, 7.7x coverage is sufficient, whereas the tetraploid, autotetraploid, and autoactoploid require 15x, 48x, and 100x, respectively, for accurate SNP genotyping by GBS. For example, the suggested sequencing depth in potatoes is 60x to avoid type I and type II errors in SNP genotyping (Clevenger et al., 2015).

The second problem for the generation of robust SNP data is the raw data analysis of the sequence reads that are generated. Data duplications, such as PCR duplication, is a common problem faced by many computational biologists (Clevenger et al., 2015). It is very difficult to categorize the natural duplication from PCR duplication, which results in incorrect InDel/SNP calling even in diploids (Li, 2014). Despite severe limitations, some of the researchers have successfully identified the SNPs through the Next Generation Sequencing (NGS)-enabled method, genotyping-by-sequencing (GBS): 20K SNPs (164 wheat DH lines), 76K SNPs (14 sugarcane accessions), and 84K SNPs (151 sugarcane clones) (Poland et al., 2012; Yang et al., 2017). The Universal Network-Enabled Analysis Kit (UNEAK pipeline) was developed as a reference method and supplemented with the TASSEL-GBS SNP calling software useful for complex genomes (Lu et al., 2013). Several studies have identified large numbers of SNP markers in sugarcane through GBS (Balsalobre et al., 2017; Yang et al., 2017) or hybridization-based target enrichment method combined with NGS (Song et al., 2016) or the generation of an Axiom SNP array (Aitken et al., 2016). Assuming the pseudo-diploid model, the heterozygous alleles of single-dose markers were scored as one group and used in the genomic prediction studies in sugarcane (Deomano et al., 2020; Hayes et al., 2021; Voss-Fels et al., 2021; Yadav et al., 2021). Though this has resolved multi-dosage effects of markers, it does not reflect the true genomic complexity of sugarcane (Voss-Fels et al., 2021) and yet the considerable improvement in genomic prediction accuracies for cane and sugar yields under the additive and non-additive genetic models is found worthy in sugarcane. Furthermore, the use of these multi-dose markers in breeding remains a challenge and there exists a further need to address the computational challenges for the utilization of multi-dose markers in complex polyploid crops like sugarcane.

### Population Structure, Genetic Diversity, and Relatedness of Training and Testing Populations

The design of the training and testing populations has to consider several parameters such as trait heritability, population structure, genetic architecture, size of the training and validation populations, and genome-wide distribution of markers. Genomic prediction accuracies are influenced by the population structure and genetic relatedness of training and testing populations (Windhausen et al., 2012; Sallam et al., 2015). The population structure arises either due to linkages between the similar alleles or genomic regions among closely related individuals or due to the linkage disequilibrium of similar genomic regions among the distantly related individuals, which does not decay over the fewer generations of selfing or crossing. Both linkage and linkage disequilibrium contribute to the population structure and requires due considerations in genomic prediction (Daetwyler et al., 2012). The minimal population structure, maximum genetic diversity among the training population and proximal genetic relatedness between training and testing populations amplified the genomic prediction accuracies (Clark et al., 2012; Daetwyler et al., 2012; Guo et al., 2014; Sallam et al., 2015). To achieve the same, several statistical approaches were used in different studies such as *k*-vertex connected graph with the maximum number of edges and the *K*-mean clustering strategy (Maenhout et al., 2010), clusters of relationship coefficients matrices (Saatchi et al., 2011), CD mean and generalized CD (Rincent et al., 2012, 2017), stratified sampling based on Euclidian distance and Ward's hierarchical clustering, CDmean, PEVmean, and stratified CDmean (Isidro et al., 2015), and the weighted additive relationship matrix with a stratified sampling (Zhang et al., 2010; Wang et al., 2018a; Berro et al., 2019).

Several GWAS studies revealed that population structure is evident in sugarcane. The population structure analysis in the panel comprising of 134 accessions of cultivars and popular parental lines displayed a very strong linkage disequilibrium with four different groups (Barreto et al., 2019). The population structure analysis in a 96 genotypes panel comprising of wild species by using the target region amplified polymorphism (TRAP) markers presented the two subgroups for sucrose metabolic pathways and three subgroups for lignin metabolic pathways. Most of the traditional cultivars, accessions of *S. spontaneum* and *Erianthus* spp. formed one subpopulation, whereas the modern cultivars were assigned into the second subpopulation (Junior et al., 2020). The population structure analysis in 97 elite and historic sugarcane varieties by using 6,534 InDel and SNP markers revealed the strong structural differentiation into two major subgroups and a stronger marker-trait correlation with sucrose traits (Fickett et al., 2019). Sugarcane crop is a photosensitive crop which requires specific environmental regimes for plant developmental activities (Baez-Gonzalez et al., 2017). The crop growth and developments differ with agro-climatic zones such as tropical and subtropical regions. The spatial variation is well-known in sugarcane and the association mapping based on a single location resulted in the identification of markers, which does not possess any significance for utilization in sugarcane breeding programmes (Wei et al., 2010). Therefore, the formation of training and testing populations has to provide due consideration to the multi-environmental evaluation and environmental covariates to enhance the genomic prediction accuracies (Pandey et al., 2020) in sugarcane.

### Trait Heritability and Genetic Architecture in Training and Testing Populations

The genetic architecture of quantitative traits describes the characteristic features of broad-sense heritability or proportion of heritable total phenotypic variation, referring to the number of genes, genomic regions, the magnitude of gene effects, and their relative contributions to the additive, non-additive and epistatic gene actions (Holland, 2007). Broad-sense heritability accounts for the total heritable variation or genotypic variation due to additive and non-additive genes. The narrow-sense heritability explains the proportion of genetic variation governed by additive genes. Broad-sense heritability is beneficial for the prediction of the genetic gain due to the selection of superior plant types in ground nursery, and narrow-sense heritability used to predict the better parental cross combination in hybridization programmes (Jackson, 2005). However, sugarcane has a long history of low narrow-sense heritability for cane yield (Wei and Jackson, 2017), a large proportion of genetic variability governed by non-additive genes (Yadav et al., 2021). Hence, parental selection based on meager breeding value does not help to enhance the genetic gain in sugarcane (Wei and Jackson, 2017). Very high broad-sense heritability was reported in sugarcane for stalk number, stalk diameter, brix, bagasse, fiber, and lignin content (Gouy et al., 2013). The make use of genome-wide markers and genomic selection aids in assessing the non-additive genetic variance in order to increase the frequency of favorable alleles in the breeding populations and selection of heterotic clones in sugarcane (Voss-Fels et al., 2021; Yadav et al., 2021).

## Genomic Selection Schemes for Sugarcane Breeding Programmes

The genomic selection schemes applied at various stages of sugarcane breeding such as germplasm characterization and core germplasm formation, pre-breeding, and genetic base-broadening programmes, selection of parents for hybridization and prediction of progeny performance, varietal development and deployment are summarized in Table 1. The feasibility and suitability of these schemes in sugarcane breeding programmes are discussed.

**Table 1:** The application of genomic prediction and genomic selection in sugarcane breeding programmes.

**Sl. no**	**Breeding programmes**	**Objectives**	**Similar studies in other crops**
1	Characterization of *Saccharum* field gene bank by using genome-wide markers	To characterize the *Saccharum* field gene bank for genomic diversity, to establish identity of accessions and to verify the duplications in gene banks.	Maize (Lu et al., 2009) Rye (Bolibok-Bragoszewska et al., 2014) Apple (Buiteveld et al., 2021)
2	Core collections of *Saccharum* field gene bank	Core sampling of *Saccharum* and allied germplasm representing the total genomic diversity present in the gene bank.	Soybean (Jeong et al., 2019) Wheat (Pascual et al., 2020) Rice (Kumar et al., 2020a)
3.	Genomic prediction in *Saccharum* gene bank	To expedite the characterization of *Saccharum* germplasm for cane yield, commercial cane sugar (CCS) and tolerances to biotic and abiotic stresses.	Sorghum (Yu et al., 2016) Wheat landraces (Crossa et al., 2016b; Kehel et al., 2020) Soybean (Peixouto et al., 2016) Cauliflower (Thorwarth et al., 2018)
4.	Genome-wide association studies and genomic prediction	To identify the genomic regions associated with agronomic traits (cane yield and CCS) and, biotic and abiotic stress tolerances.	Eucalyptus (Müller et al., 2017) Winter wheat (Kristensen et al., 2019; Odilbekov et al., 2019) Wheat and Barley (Tsai et al., 2020) Maize (Liu et al., 2021)
5.	Pre-breeding or wide hybridization or nobilization	To introgress new alleles and genomic regions from *Saccharum* germplasm identified through germplasm characterization, GWAS and genomic prediction studies into the working germplasm.	Maize (Gorjanc et al., 2018) Eucalyptus (Tan et al., 2017) Pear (Kumar et al., 2019)
6.	Genetic base-broadening	To improve the breeding value of the genetic stocks identified from pre-breeding by backcrossing with parental lines or recurrent selection	Eucalyptus (Tan et al., 2017) Wheat (Singh et al., 2018) Pear (Kumar et al., 2019) Maize (Mayer et al., 2020)
7.	Recurrent selection breeding programmes (Gouy et al., 2013)	To augment the favorable alleles in the population/parental pool for cane yield, CCS, and tolerances through recurrent selection cycles	Rice (Grenier et al., 2015) *Brassica napus* (Zhao et al., 2016) Maize (Müller et al., 2017) Blueberry (Ferrão et al., 2021)
8.	Genomic prediction of parental cross combination and hybridization	Prediction of parental cross/progenies combination through estimated breeding values, general and specific combining ability of parental lines	Apple (Kumar et al., 2020b) Maize (Kadam et al., 2016; Jarquin et al., 2021; Liu et al., 2021) Wheat (Lado et al., 2017) Cassava (Wolfe et al., 2017)
9.	Progeny assessment and clonal selection (Deomano et al., 2020; Hayes et al., 2021; Yadav et al., 2021)	Prediction of superior plant types/progenies based on the broad-sense heritability or additive and non-additive genetic variance	Cassava (Wolfe et al., 2017) Eucalyptus (Resende et al., 2017) Sugarcane (Deomano et al., 2020; Hayes et al., 2021; Yadav et al., 2021) Oil palm (Nyouma et al., 2020) Macadamia nut (O'Connor et al., 2021)
10	Multi-environment trial or deployment of cultivars to target environments	To predict the genotype × environment interactions in multi-environment trials and to identify the stable varieties suitable for the target environment	Barley (Oakey et al., 2016) Chick pea (Roorkiwal et al., 2018)
		To predict the marker × environment interaction in multi-environment trials and identify the environment sensitive genomic regions	Maize (Schulz-Streeck et al., 2013) Wheat (Lopez-Cruz et al., 2015; Crossa et al., 2016a) Rice (Monteverde et al., 2019)
		Incorporation of environmental covariates into the genomic models and to predict the impact of environmental covariates on genotype performance and deployment of varieties.	Wheat (Heslot et al., 2014) Barley (Malosetti et al., 2016) Rice (Monteverde et al., 2019)

### Genomic Prediction and Core Collection in Field Gene Banks

The large number of germplasm collections maintained at gene banks have issues of germplasm duplication, the constraint of rejuvenation of a large number of accessions, unsatisfactory phenotyping, and utilization in breeding programmes (Díez et al., 2018). The core set formation is highly significant in resolving the problems of duplications and the ideal core collection is composed of 10% of the entire germplasm collection, representing 70% of the alleles of the entire germplasm (Brown, 1989). The various strategies such as stratified random sampling (Brown, 1989) and sampling based on multivariate clustering (Franco et al., 2005) were used in the core collection of germplasm. The molecular markers were also used in core germplasm formation in various crops (Zaharieva et al., 2001; Hao et al., 2006; Krichen et al., 2012; Dutta et al., 2015; Liu et al., 2015). The advent of next-generation sequencing technologies (NGS) and reduction in the cost of sequencing have given a new avenue for genome-wide SNP discovery and their utilization in genomic prediction in germplasm and core germplasm. Genome-wide marker-based germplasm characterization and core collection are validated in crops like wheat (Crossa et al., 2016b; Kehel et al., 2020), sorghum (Yu et al., 2016), soybean (De Azevedo Peixoto et al., 2017), and cauliflower (Thorwarth et al., 2018).

The global collections of sugarcane germplasm are maintained at World Collections of Sugarcane and Related Grasses (WCSRG), Canal Point, Florida, and the Indian Council of Agriculture Research (ICAR)-Sugarcane Breeding Institute, Coimbatore, India. About 1,002 accessions and 3,345 accessions of *Saccharum* germplasm are maintained at Canal point and Coimbatore, respectively (Amalraj and Balasundaram, 2006; Nayak et al., 2014). Besides, wild relatives are also maintained in Fiji, Brazil, Australia, China, and many other countries and are actively being used in the breeding programme (Ramdoyal and Badaloo, 2002; Wang et al., 2008; da Silva, 2017; Bhuiyan et al., 2019; Cursi et al., 2021). Several studies of core collection in *Saccharum* germplasm are described based on morphological features and molecular markers (Balakrishnan et al., 2000; Tai and Miller, 2001; Balakrishnan and Nair, 2003; Amalraj et al., 2006; Nayak et al., 2014; Shadmehr et al., 2017; Tena Gashaw et al., 2018; Fickett et al., 2019). The phenotypic characterization of *Saccharum* germplasm has many hurdles. First, tall and long duration crops are more prone to lodging and sucker development, which directly influences biomass and cane yield and juice quality parameters (Berding et al., 2005). Second, sugarcane is a photosensitive crop and spatial variation or morphological expressions are influenced by specific environmental regimes (Waldron et al., 1967; Gosnell, 1973; Pereira, 1983; Shanmugavadivu and Rao, 2009; Wei et al., 2010). Third many countries prohibited the field evaluation of *S. spontaneum* germplasm which are classified as noxious weed due to their fast-growing, spreading type with rhizatomous roots (Todd et al., 2017). Fourth, morphological characterization of germplasm based on fewer morphological descriptors and environmentally sensitive quantitative data does not reflect the complete genetic variability of *Saccharum* germplasm and unlike the utilization of genome-wide markers that helps to capture the complete genetic variability of genetic resources (Nybom and Lācis, 2021). Therefore, germplasm characterization and the formulation of core germplasm by using genome-wide markers could be the best option for crops like sugarcane which additionally captures the complete genetic variability.

In-depth pedigree analysis of the sugarcane germplasm has shown that only a limited number of basic *Saccharum* species clones were used in the development of breeding lines in sugarcane (Jackson, 2005; Kumar et al., 2012). These breeding lines were further advanced through recurrent selection and utilized in the varietal developmental programmes (Jackson, 2005). The pedigree analysis of Indian “Co” varieties (Figure 1) showed that only two *S. spontaneum* accessions *viz., S. spontaneum* CBE and *S. spontaneum* Java, were used in the breeding programmes (Kumar et al., 2012). Although there were efforts made to utilize the *Saccharum* germplasm in sugarcane hybridization programme for pre-breeding and base-broadening programmes (Wang et al., 2008; Mohanraj and Nair, 2014; da Silva, 2017; Nair et al., 2017; Cursi et al., 2021), still it was not a complete utilization of *Saccharum* germplasm or all the favorable alleles contributing to the cane yield and sucrose. Therefore, characterization of the *Saccharum* germplasm with genome-wide markers and genome-wide association studies certainly identifies the alleles contributing to the cane yield, sucrose content, and other agronomic traits. The genome-wide markers capture the complete genetic variability present in germplasm and are also useful in the formulation of core germplasm, improving the precision of selection in gene-specific introgression in pre-breeding and enriching the favorable alleles and genes in genetic base-broadening and recurrent breeding programmes. The two major concerns of a sugarcane breeder, as described by Jackson (2005) *viz.*, the concern of narrow genetic base and potential opportunities to broaden the genetic base by utilizing a large number of basic *Saccharum* germplasm, were easily addressed by adopting the genomic prediction in *Saccharum* gene bank and genomic selection schemes in pre-breeding and genetic base-broadening programmes. As described in sorghum (Yu et al., 2016), *Saccharum* germplasm can be used for genomic prediction by considering the part of the germplasm as the training population and the remaining germplasm as the validation population.

### Genomic Selection for Pre-breeding and Genetic Base-Broadening Programme

Pre-breeding and genetic base-broadening is the most important component of the sugarcane breeding programme followed in many countries (Ramdoyal and Badaloo, 2002; Wang et al., 2008; Matsuoka et al., 2014; da Silva, 2017; Cursi et al., 2021). The importance of *S. spontaneum* and other *Saccharum* spp. in the development of cultivars for bioenergy and commercial sugar production has been previously reviewed (Wang et al., 2008; Matsuoka et al., 2014; da Silva, 2017; Cursi et al., 2021) and novel genetic resources for disease resistance were identified (Bhuiyan et al., 2019). The utilization of *S. spontaneum* accession “Mandalay” in varietal development programmes resulted in many varieties in Australia (Reffay et al., 2005; Piperidis et al., 2021) and the high yield sugarcane cultivar LCP 85-384 has genetic lineage from *S. spontaneum* and *S. barberi* (Milligan et al., 1989). The studies involving genotyping of 400 markers in 232 biparental populations, which were derived from crosses involving Mandalay as a grandparent, exhibited that 25% of genomic regions originated from the “Mandalay” (Reffay et al., 2005). Consistent efforts were made to utilize wild genetic resources to broaden the genetic base of Indian working germplasm involving many *Saccharum* spp. such as *S. officinarum, S. robustum, S. spontaneum* as well as *E.arundinaceus*, and *Erianthus bengalense* (Mohanraj and Nair, 2014; Ravinder et al., 2015; Nair et al., 2017). Nevertheless, phenotypic selection might not have captured the complete favorable alleles. Furthermore, the utilization of genome-wide markers, genome-wide association studies, and genomic selection helped in enhancing the frequency of favourable alleles and minimising the linkage drag, which are commonly associated with wide-hybridization and genetic base-broadening programmes in sugarcane (Roach, 1989).

**Figure 2:**
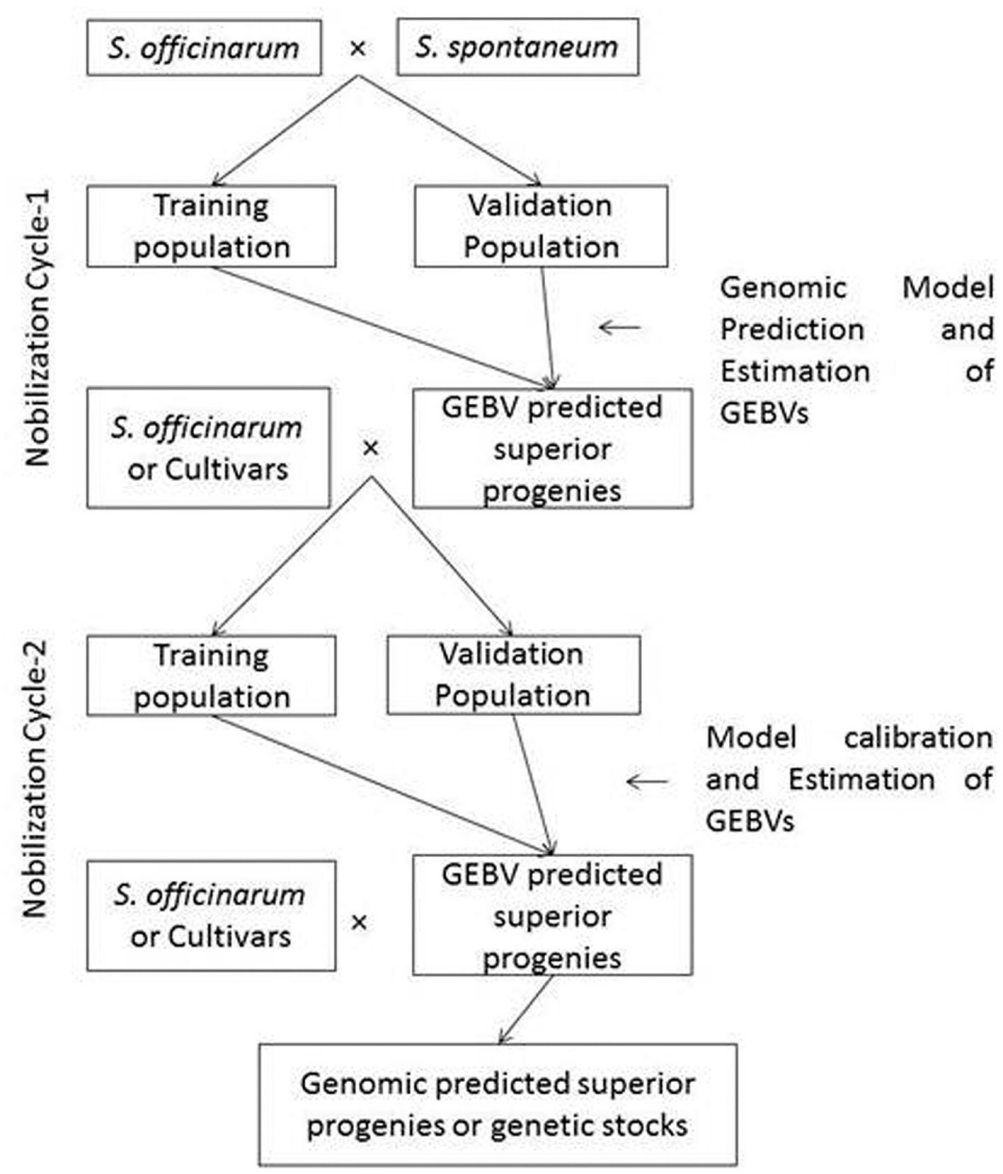
Genomic selection schemes for pre-breeding and genetic base-broadening programmes in sugarcane. The sugarcane pre-breeding requires three or more number of backcrossing with recurrent parents. Wild relatives such as *S. spontaneum, Saccharum robustum*, and *S. barberi* are used as male parents and *S. officinarum* or improved “Co” canes are used as female parents. Cycle 1 requires hybridization between *S. officinarum* or “Co” canes with wild species and Cycle-2 requires backcrossing progenies derived from cycle-1 with *S. officinarum* or “Co” canes. The genomic selection schemes are required to be applied in both cycle-1 and cycle-2. The true F_l_ at Cycle-1 identified by molecular markers and genomic models are required to train at every backcrossing to improve the genomic prediction accuracy.

Pre-breeding, a bridge between crop improvement and plant genetic resources, provides an opportunity for introgression of desirable genes with minimal linkage drag and enhances the adaptability of the cultivars (Sharma et al., 2013). It is combined with genomic-assisted selection which is helpful in the identification of climate-smart alleles/haplotypes in gene banks and in the development of climate-resilient varieties (Varshney et al., 2018). Moreover, it was also demonstrated in other clonally propagated crops (Tan et al., 2017; Kumar et al., 2019). In similar ways, the feasibility of genomic prediction in sugarcane pre-breeding has to be explored and the methodology is described in Figure 2. The pre-breeding in *Saccharum* spp. requires 3–4 generations of backcrossing with recurrent parents. Pre-breeding in sugarcane involves the following steps: (i) genome-wide association studies in germplasm and identification of elite accessions with more number of desirable genes/trait-specific accessions; (ii) hybridization between elite accessions with noble *S. officinarum* clones or commercial sugarcane varieties; (iii) identification of true interspecific hybrids through genomic/cytoplasmic/5S rRNA/Inter Transcriber Spacer specific markers; (iv) optimization of the training and testing populations at each generation or back cross programmes which generally requires 3–4 generations of backcrossing/crossing with commercial sugarcane varieties; (v) genomic prediction model building and retraining of the genomic prediction models to optimize and predict the genotype with the highest Genomic Estimated Breeding Value (GEBV) for utilization in the next generation of backcrossing or crossing. The genomic prediction model could also be able to help in the swift development of genetic stocks in pre-breeding and genetic base-broadening in sugarcane.

### Genomic Selection for Superior Parental Cross Combination and Clonal Selection at an Early Stage

The varietal development programme in sugarcane breeding is a kind of recurrent selection. The parental lines for hybridizations are selected based on breeding values estimated from the performance of families or progenies of proven crosses (Chang and Milligan, 1992; Jackson, 2005; Burnquist et al., 2010; Lingle et al., 2010; Stringer et al., 2010; Mendes de Paula et al., 2020). Sugarcane is a clonally propagated crop, amenable for the exploitation of both additive and non-additive gene action. The additive genetic variance determines the proportion of genetic variability transmitted to progenies or breeding value. This is helpful in the selection of an elite parental pool and prediction of parental cross combinations. The proportion of non-additive genetic variance is suitable for the selection of elite plant types from the ground nursery (Jackson, 2005; Mendes de Paula et al., 2020). A very high significant Specific Combining Ability (SCA) variance is observed for cane yield in an unselected population of 35 families indicating the predominance of non-additive genetic variance (Zhou, 2020). The evaluation of 79 families derived from 38 parental lines revealed the predominance of non-additive genetic variance for cane yield and additive genetic variance for brix and sucrose (Mendes de Paula et al., 2020). Sugarcane has a long history of low narrow-sense heritability and a higher proportion of non-additive genetic variance for cane yield as compared with CCS (Wei and Jackson, 2017; Mendes de Paula et al., 2020; Zhou, 2020; Yadav et al., 2021). Hence, the selection of parental lines based on breeding value does result in a low genetic gain and require due consideration for non-additive genetic variance in the choice of a parental pool for hybridization programmes.

The breeding programme in sugarcane is aimed at enhancing the genetic gain for CCS and cane yield. There is no strong correlation between cane yield and CCS. The absence of a large correlation between the cane yield and CCS indicates that either gene governing these traits are independent or acting pleiotropically (Jackson and McRae, 2001; Zaharieva et al., 2001). Both traits are polygenic traits, governed by many genes with minor effects (Hoarau et al., 2002; Ming et al., 2002; Aitken et al., 2008), and the genetic gain through phenotypic selection is low, as the probability of progenies with combinations of superior alleles for sucrose and cane yield is very low (Jackson, 2005). The recurrent selection combined with family evaluation increases the probability of the accumulation of favorable alleles in the population and the chances of selecting the superior plant types combined with both cane yield and CCS is high (Chang and Milligan, 1992; Lingle et al., 2010). The genomic prediction schemes are extensively used in other crops for estimation of non-additive genetic variance and prediction of the best of parental cross combination based on their general and specific combining ability variances and effects (Kadam et al., 2016; Lado et al., 2017; Wolfe et al., 2017; Jarquin et al., 2020; Wang et al., 2020). The encouraging results of genomic prediction in recurrent selection schemes are also reported in many crops (Zhao et al., 2016; Ferrão et al., 2021). Recently, the genomic selection for recurrent selection schemes suitable to sugarcane was reviewed (Yadav et al., 2020) and encouraging results for estimating the breeding values based on additive genetic variance with high prediction accuracies are demonstrated (Gouy et al., 2013; Deomano et al., 2020; Hayes et al., 2021). The simulation and empirical research have demonstrated the significant contribution of non-additive genetic variance and genome-wide heterozygosity for cane yield and CCS in sugarcane (Voss-Fels et al., 2021; Yadav et al., 2021).

At present, the parental cross combination in sugarcane is selected based on breeding values and past proven crosses. A large number of crosses are made and 30–50,000 of progenies are evaluated for identification of superior plant types combined with high cane yield, CCS, and resistance to many diseases like smut, red rot, and yellow leaf disease and tolerances to abiotic stresses (Park et al., 2007; Nair, 2011). The clonal trials are evaluated in single row experiments and advanced trials in multiple row experiments. The evaluation and selection in the ground nursery and clonal trials have many technical and logistics problems. First, a large portion of genetic variability for CCS is governed by additive genes and there is a high correlation between early and advanced clonal selection cycles. For cane yield, the non-additive genetic variance contributes significantly to the genetic variance and is highly influenced by environmental factors. The correlation between the smaller and larger plot experiments is very low for the cane yield as compared with CCS due to field competitive effects. Hence, the selection of clones in the ground nursery and early clonal trials are less reliable (Jackson and McRae, 2001). Second, the lodging and suckering propensity in the ground nursery and clonal trials are major hurdles for breeders in choosing the superior plant types. Third, trait measurement for sucrose content varies with a sound cane, i.e., millable cane developed from first formed tillers, and non-sound canes, i.e., millable canes developed from late emerged tillers. For example, CCS is highly variable with sound and non-sound canes (Berding et al., 2005; Jackson, 2005). Fourth, the crop expression varies with agro-climatic regions, such as tropical and subtropical regions and the location-specific ground nursery essentially requires for identification of location-specific varieties (Park et al., 2007; Nair, 2011). Considering all these factors, genomic selection could assist the breeders in the selection of potential genotypes that combine with the high cane yield, CCS, and tolerance to biotic and abiotic stresses. With the accurate multi-environment phenotyping of the parental pool and populations derived from recurrent selection schemes, the genomic prediction could help in identifying the plant types suitable to different agro-ecological regions and even a common phenotype suitable across zones. Additionally, it helps in reducing the population size to be evaluated in the ground nursery and subsequent clonal trials. Genomic selection is a evolving science, and genomic prediction models fitted with additive and non-additive components and genome-wide heterozygosity has demonstrated its efficiency in sugarcane (Gouy et al., 2013; Deomano et al., 2020; Hayes et al., 2021; Voss-Fels et al., 2021; Yadav et al., 2021). Further developments in data science, training, and retraining of genomic statistical models will impart benefits to the sugarcane breeders in the selection of the superior cross combinations and varieties in sugarcane.

### Genomic Predictions for Environment Covariates and Varietal Deployment

The genotypes developed from the breeding programmes are adapted to the specific environments and the differential performance of genotypes in different environments is due to genotype × environment (G × E) interactions. The repeatable G × E factors in multi-environment trials are estimated through single dimension based statistical models such as ANOVA and regression approaches. The ANOVA based statistical model partitioning the G × E interactions into the main effect of genotypes, environments and their interactions as fixed effects/variables and residuals/error components as random variables (Elias et al., 2016). The linear regression-based G × E interaction (Finlay and Wilkinson, 1963) introduce the slopes for environmental means and identifies the genotypes based on the slope and trait mean value. The G × E interactions caused by several factors (Gauch, 2006) and statistical models *viz.*, additive main effects and multiplicative interaction effects model (AMMI), site regression model (SREG), shifted multiplicative model (SHMM), genotype regression model (GREG), and completely multiplicative model (COMM), are used for partitioning of the G × E interactions into more than one factor (Elias et al., 2016). The linear and multiplicative models that treat the genotypes and environments as fixed effects do not consider the heterogeneity of variance. They are suitable only for the balanced dataset as they do not consider the variable replication numbers, which can be accommodated under mixed effect models.

The mixed model framework permits the utilization of correlated heterogeneous variance under the variance-covariance structure of random variables and estimates the association between known environmental and genetic parameters. Factor regression is used for the analysis of multi-environmental trials and statistically helps to test the sensitivity of genotypes with environmental covariates (Gauch et al., 2008). The partial least square regression analysis on G × E interaction allows the use of environmental covariates as independent variables to find the most influential environmental variables contributing to the interactions (Vargas et al., 1998). The factorial regression analysis offer aid in integrating the environmental covariates into the model and determine the most influential environmental parameters affecting the trait/QTL expression (Malosetti et al., 2004).

The genomic prediction models can be applied to multi-environment trials, which require characterization of G × E interactions over locations, environments and years. The covariance structures of markers, pedigree, environmental covariates, marker × environmental covariates are incorporated into the genomic models to predict the G × E interactions, environmental sensitivity of the genotypes, and identify the environment-sensitive genomic regions/QTL contributing to the G × E interaction and assessing the prediction accuracy in untested environments (Crossa et al., 1999; Burgueño et al., 2012; Schulz-Streeck et al., 2013; Heslot et al., 2014; Montesinos-López et al., 2016; Oakey et al., 2016; Monteverde et al., 2019). Multiple environment-genomic prediction models demonstrated the prediction of the genome-wide environment-specific genomic regions and the model envisaged the environmental covariates associated with phenology and crop growth models (Malosetti et al., 2016).

The differential response of genotypes to different environments is highly evident in sugarcane such as: (i) sandy soils have high discriminative powers in differentiating the environments and genotypes (Glaz and Kang, 2008); (ii) multi-environment trials involving seven locations revealed the significance of genotype × location interaction, which is higher than genotype × crop year interaction and inferred that testing genotypes across locations are more important than repeating the trials in the same locations (Guilly et al., 2017); (iii) time of ratooning/planting and harvesting season contributes significantly to the G × E interactions for cane yield and sugar yield (Gilbert et al., 2006), partly due to the non-repeatable interactions (Ramburan, 2014); (iv) spatial variation also significantly contributes to the G × E interactions in sugarcane (Wei et al., 2010). Therefore, multi-environment trials play a crucial role in the selection of stable region-specific better-performing varieties suitable for cultivation across locations. In the Australian sugarcane varietal development programme, the final stage of clonal selection is chosen through evaluation in the final assessment trials conducted in four agro-climatic zones *viz.*, Northern, Burdekin, Central, and Southern regions (Park et al., 2007). The Louisiana varietal identification programme involves replicating the Outfield Variety Tests in many locations (Breaux, 1984). The Brazilian breeding programme under Rede Interuniversitária para o Desenvolvimento do Setor Sucroenergético (RIDESA, Brazil) network requires assessment in multiple locations and harvests in the final phase of recommendation of clones (Barbosa et al., 2012). In India, sugarcane growing regions are broadly categorized into five different agro-climatic regions or zones, namely Peninsular, North-West, North-East, North-Central, and East-Coast regions, wherein each zone has its multi-environment trials for final varietal identification (Nair, 2011). The implementation of genomic schemes by integrating with environment covariates for the prediction of clonal performance in each agro-climatic zones certainly helps in the identification of the environment-specific genomic regions and superior clones combined with cane yield, CCS, and tolerances to biotic and abiotic stresses. The genomic prediction for clonal performance in different locations is validated for cane yield, CCS, and fiber content with high prediction accuracy (Yadav et al., 2021) and further research is required to address the challenges of genomic modeling to accommodate more parameters related to environmental parameters.

## Genetic Gain Per Unit Cost and Unit Time, and Resource Allocation

The genomic selection reduces the number of selection cycles and reduce the duration of the breeding cycles. The genetic gain in breeding has to be assessed in terms of gain per unit time and cost rather than the gain per cycle (Bernardo, 2008). The superiority of genetic gain due to genomic selection over the phenotypic selection and marker-assisted selection with the reduced cost and duration of selection was demonstrated in many crops (Bernardo and Yu, 2007; Wong and Bernardo, 2008; Yabe et al., 2018). The genomic selection minimizes the phenotyping and maximizes the genotyping and is worth considering when the phenotyping cost is much higher than genotyping (Bernardo and Yu, 2007). In Indian conditions, the cost of genotyping through 50K SNP chips and genotyping by sequencing is around Rs. 10,500.00 per sample. The cost of genotyping in a ground nursery or progeny assessment trials, which accommodates 12,000 seedlings per ha costs about Rs. 12.60 crores, the First Clonal Trial (4,800 clones/ha) costs around Rs. 5.04 crores, Second Clonal Trial (1,200 clones/ha) costs around 1.26 crores, Pre-Zonal Varietal Trial (150 entries with two replications per ha) costs around Rs.15.75 lakh, and AICRP Trials (50 entries with three replications per ha) costs around Rs. 5.25 lakhs. Therefore, the genotyping cost in India is still higher and highly expensive as compared with phenotypic selections and similar reports of the higher genotyping cost of the SNP array ($95/sample) was also reported in Australia (Voss-Fels et al., 2021). Yet, the superiority of genetic gain through genomic selection as compared with phenotypic selection is significantly high in terms of net return and scientifically embracing to implementation of the genomic selection at the progeny assessment stage (Beyene et al., 2019; Voss-Fels et al., 2021). The genomic-wide selection for the estimation of GEBVs for germplasm, lines derived from pre-breeding, parental lines, progeny performance in progeny assessment trials and multi-environment trials is essentially required to enhance the genetic gain in sugarcane.

Optimization and efficient resource allocations in the genomic selection are necessary for improving genetic gain and prediction efficiency. Strategies for resource allocation depends on the role of genomic selection in breeding population, such as (i) size, composition and design of training and validation populations, (ii) plot size for breeding experiments varies with stages of breeding programmes, such as parental selection, early and advanced testing, and (iii) the number of replications within and across the environments (Lorenz and Nice, 2017). The composition of the training population is determined based on the objective of genomic selection schemes. The training and validation populations could be diverse germplasm, elite parental lines of diverse origin, segregating breeding lines from the same cross or diverse biparental population, early testing of breeding lines and multi-environment trials (Windhausen et al., 2012; Yu et al., 2016; Roorkiwal et al., 2018; Ozimati et al., 2019). Moreover, each study has its significance in plant breeding and demands different strategies for resource allocation.

The genomic selection in the early stages of segregating the population is a trade-off between the cost of genomic selection and trait heritability and family size. The phenotypic selection in a large segregating population with highly heritable traits is highly efficient, whereas low heritable traits, such as yield in a large segregating population, require the molecular markers to assist in the selection of desirable plant types. Further, yield measured on the single plant basis in segregating population is inaccurate in most of the crops (Heslot et al., 2015). Even after the selection of elite plant type based on GEBV, phenotypic evaluation is needed to remove the inferior genotype if any developed due to the large allelic mutation or rare alleles becoming homozygous (Hayes et al., 2009). Considering the crop duration, crop architecture, and other field related problems, such as lodging and suckering propensity, variation in trait value due to sampling errors in sound and non-sound canes, field competitive effects (Jackson and McRae, 2001), the simulation studies demonstrated the economic benefits of implementing the genomic selection in sugarcane (Voss-Fels et al., 2021), and genomic prediction accuracies are also experimentally validated (Yadav et al., 2021).

The recurrent selection procedure is effective in augmenting the frequency of favorable alleles in the breeding population. The marker- or genomic-assisted selection helps to reduce the number of breeding cycles and the number of individuals evaluated in each selection cycle. The marker-assisted recurrent selection scheme aims at gene pyramiding after identifying the QTL (Servin et al., 2004), whereas the genomic assisted recurrent selection scheme aimed at gene pyramiding without identification of QTL (Bernardo and Yu, 2007; Bernardo, 2009). The genomic-assisted recurrent selection could reduce the prediction accuracies after several cycles (Muleta et al., 2019) and requires genomic remodeling at each cycle of selection, which may add to cost (Heslot et al., 2015). The marker-assisted selection schemes operate with fewer markers on the biparental population while the other operates with a large number of markers, further adding to the cost (Heslot et al., 2015). Even after the selection of elite plant type by using GEBVs, phenotypic evaluation is needed to remove the inferior genotype if any developed due to the large allelic mutation or rare alleles becoming homozygous (Hayes et al., 2009). The genomic assisted recurrent selection accelerates the genetic gain over a shorter period in both small and larger populations. The cost per unit gain is lower for oligogenic traits in smaller populations and polygenic traits in large populations as compared with phenotypic recurrent selection (Muleta et al., 2019). The recurrent selection cycles in sugarcane operate in two ways. First, the selection of ideal plant type from the ground nursery, vigorous testing for the identification of promising clones and further utilization in hybridization programme forms a kind of random recurrent selection cycle. Second, the 2–3 recurrent selection cycles maximize the genetic gain or trait values (Shanthi et al., 2008; Yadav et al., 2020), and the simulation study has validated the cost-benefit ratio and resource allocation to maximize the genetic gain (Voss-Fels et al., 2021).

The multi-environment trials are the final phase of cultivar identification and need robust statistical analysis with optimum resource allocation. The resource allocation depends on the number of replications, locations, number of test entries, etc. An adequate number of replications are required for each location to control the microenvironment variations and experimental design such as Alpha design, which is very efficient in accounting for the micro-environment variation (González-Barrios et al., 2019). The optimal number of replications and test locations require optimization based on historical data and the number of mega environments estimated from genotype + genotype × environment (GGE) biplot analysis (Yan, 2015; Yan et al., 2015). The four replications were found optimum based on GGE biplot analysis of multi-environmental trials (Baxevanos et al., 2017). The very high significant genotype × location interactions and very low interactions for genotype × crop and genotype × year components were observed. Based on the ranking of genotypes from all replicates and two replicates in multi-environment trials, inferred that two replications did not affect the precision of selections and save 33–50% of the experimental area (Yan, 2021). The extremely low variance for genotype × replication interactions was observed and a reduction in the number of replications from eight to four did not reduce the precision of the experiments in sugarcane (Brown and Glaz, 2001). The historic mega-environments are useful in differentiating the genotypes and helps in resource allocation. The moderate mega-environment are highly useful to achieve the optimum genetic gain (González-Barrios et al., 2019).

The unbalanced design or sparse testing refers to the multi-environment trials where all the genotypes are not tested in all environments and sparse testing saves the resources. Unbalanced sparse testing with an appropriate genomic prediction model enhances the prediction accuracies and saves the resources (Endelman et al., 2014; González-Barrios et al., 2019; Jarquin et al., 2020). The resource allocation depends on many factors such as the genetic relationship of the training population, genomic prediction model calibration, target environments, number of replications, and many other factors (Lorenz and Nice, 2017). Several factors such as complex polyploid and its impact on genotyping, crop agronomy, crop architecture, crop duration, biomass, sucrose accumulation pattern, and other parameters necessitate consideration for genomic selection. The empirical studies are required to develop a comprehensive theoretical framework on resource allocations in genomic selection in sugarcane.

## Conclusions

Sugarcane is a C4 crop with a great potential for high biomass production and a major source of raw material for sugar production and bioenergy. Its crop improvement has many bottlenecks such as the long cycle of breeding duration, complex polyploidy, high degree of heterozygosity, narrow genetic base, and fewer basic germplasm utilized in pre-breeding and linkage drag during wide hybridization and limited financial and manpower resources. The genomic selection schemes are highly helpful in reducing the duration of breeding cycles, population size, and selection of desirable parents and development of varieties. The characterization of *Saccharum* germplasm with genome-wide markers captures the total genomic diversity of gene bank and assists in the formation of core germplasm and genomic prediction to identify the genes associated with cane yield, CCS, and tolerances to biotic and abiotic stresses. The genomic selection in pre-breeding and genetic base-broadening programmes helps in the precise introgression of genes into the parental clones and genomic-assisted recurrent selection is useful in augmenting the favorable alleles in the population. The genomic prediction for characterization of parental clones for their general and specific combining ability leverage the breeders in selecting the elite parents and progenies combined with favorable alleles for cane yield, CCS, and tolerances to biotic and abiotic stresses. The integration of environmental covariates into genomic models predicts the better performing varieties for target environments and deployment of varieties. Genomic selection is an evolving science; appropriate genomic models and breeding strategies strengthen the prediction accuracies and enhance the genetic gain in sugarcane.

## Author Contributions

CM conceptualized, wrote, and reviewed the draft. KA wrote, revised, provided inputs on molecular markers, SNPs, genomic selections, and resource allocations. CA, GS, PV, HM, RV, GH, GA, and BR provided inputs on molecular markers, genomic schemes, and resources allocations. All authors read and approved the manuscript.

## Conflict of Interest

The authors declare that the research was conducted in the absence of any commercial or financial relationships that could be construed as a potential conflict of interest.

## Publisher's Note

All claims expressed in this article are solely those of the authors and do not necessarily represent those of their affiliated organizations, or those of the publisher, the editors and the reviewers. Any product that may be evaluated in this article, or claim that may be made by its manufacturer, is not guaranteed or endorsed by the publisher.

## Abbreviations

CCS: Commercial cane sugar
QTL: Quantitative trait loci
NGS: Next-generation sequencing technologies
GBS: Genotyping by sequencing
SNP: Single nucleotide polymorphism
GWAS: Genome-wide association studies
BLUP: Best linear unbiased prediction
RR-BLUP: Ridge regression BLUP
LASSO: Least absolute shrinkage and selection operator
GBLUP: Genomic BLUP
RKHS: Reproducing kernel Hilbert space
DArT: Diversity arrays technology
taBLUP: Trait associated BLUP
sBLUP: Super BLUP
cBLUP: Compressed BLUP.
